# Age at Menarche and Factors that Influence It: A Study among Female University Students in Tamale, Northern Ghana

**DOI:** 10.1371/journal.pone.0155310

**Published:** 2016-05-12

**Authors:** Evans Paul Kwame Ameade, Helene Akpene Garti

**Affiliations:** 1 Department of Pharmacology, School of Medicine and Health Sciences, University for Development Studies, Tamale, Ghana; 2 Department of Community Nutrition, School of Allied Health Sciences, University for Development Studies, Tamale, Ghana; Indiana University, UNITED STATES

## Abstract

**Introduction:**

Age at menarche reflects the health status of a population. This marks the beginning of sexual maturation and is affected by nutritional status and prevailing environmental conditions. This study measured the menarcheal age of female undergraduate students in northern Ghana and explored factors that could impact on the onset of menarche.

**Method:**

GraphPad 5.01 was used to analyze data collected from 293 randomly selected female university students in a cross-sectional study using a semi-structured questionnaire. Association between different variables was tested using appropriate statistical tests.

**Results:**

The mean recall age at menarche of participants in this study was 13.66 ±1.87 years for a female population of mean age, 23.04±5.07 years. Compared to female students who lived in rural settings, urban and suburban areas dwellers significantly recorded earlier menarche (p = 0.0006). Again, females from high income earning families experienced menarche earlier than those who were born to or lived with lower income earners (p = 0.003). Lower menarcheal age increased risk of experiencing menstrual pain prior to menses rather than during menstrual flow for dysmenorrhic females. (13.52±2.052 vrs 13.63±1.582 year; χ^2^ = 7.181, df = 2, p = 0.028).

**Conclusion:**

Mean menarcheal age of female university students in northern Ghana was 13.66 years. Females from urban areas and high income families had earlier menarche. Compared to the very first Ghanaian study reported in 1989, the menarcheal age decline was 0.11 year per decade.

## Introduction

There are several milestones in the life of a girl child as she grows to become a female able to reproduce. The last major event of this sexual development is the first episode of menstrual blood flow referred to as menarche [1]. This important developmental milestone in females has been found to vary greatly across countries [2]. A study in the United States of America (US) showed that there can be variability of menarcheal age even in the same country. In this US study, it was found that the mean age at menarche among US girls in the 2000s was 12.34 years, with racial variations; 12.06 years among non-Hispanic black girls, 12.09 years in Mexican American girls and 12.52 years in non-Hispanic white girls [3].

The variation in menarcheal ages recorded across the world could be attributed to the study participants as well as the measurement instrument [4]. However, several factors have been found to significantly influence menarcheal age and they include genetics, environmental conditions, body stature, family size, body mass index, socioeconomic status, and the level of education [1], [5], [6], [7], [8], [9], [10].

Secular trend shows that age at menarche is declining in most countries. However, in developed countries which have lower menarcheal ages, there have been little or no changes in these values for a long time [9], [11], [12]. Improvement in sanitation, better nutrition as well as improved socio-economic status and better healthcare provisions in several countries are possible factors responsible for the decline or stabilization of the age at menarche [9], [13].

The current declining and earlier menarche being experienced across the world has been linked to increased prevalence of increased body mass index, insulin resistance as well as unhealthy lipid profile culminating in higher risks of cardiovascular diseases such as hypertension, coronary heart disease, strokes and diabetes in women [14], [15], [16]. Furthermore, women who experience menarche before 12 years have 23% higher risk of developing breast cancer than those who first menstruate at 15 years or more [17], [18]. Late menarche on the other hand presents with its own health burden since it has also been associated with osteoporosis, depression and social anxiety problems [9].

The first recorded study on menarcheal age of the Ghanaian female was reported by Adadevoh, Agble, Hobbs, and Elkins in 1989 which was followed by other studies by Adanu et al., (2006) and Aryeetey et al., (2011)[6], [19], [20]. Whereas the work done by Adadevoh et al., (1989) was in central Ghana, all others were conducted in Accra and its environs in southern Ghana. There is currently neither a nationwide study nor a study conducted in the northern part of Ghana to determine the age at menarche of girls. This study conducted among randomly selected female students of the University for Development Studies in Tamale, is therefore the first to determine the age at menarche of females in northern Ghana using the recall method. Considering the wide socio-economic and cultural differences between the southern and northern populations in Ghana, the study also evaluated the effect of socio-economic status and menstrual characteristics on the onset of menarche in this Ghanaian female population.

## Method

### Informed consent

Respondents’ informed consents were obtained by stating in the introductory section of the questionnaire that accepting to participate and completing the questionnaire indicated consent and that respondents had the option to withdraw at any point in the research.

### Ethical consideration

Prior approval for this study was obtained from the Ethics Committee of the School of Medicine and Health Sciences of the University for Development Studies.

### Study design and setting

This cross sectional study conducted between March and April, 2015 was among female undergraduate students of Schools of Medicine and Health Sciences and Allied Health Sciences of the University for Development Studies, Tamale, pursuing degree programmes in Medicine, Nursing, Midwifery, Health Science Education and Community Nutrition. The study sample was 389 out of a study population of 990. The instrument for this study was a semi-structured questionnaire. The questionnaire was initially piloted among 20 students which ensured correction of ambiguous and inconsistent questions before it was administered for the actual data collection. Of the 389 questionnaires distributed, 293 were returned and completed well enough to be included in this study. The response rate was therefore 75.3%

### Study size determination and sampling procedure

Using the Cochran’s correction formula for categorical data, the required sample size was calculated. Assuming 50% of respondents’ recall age at menarche was equal to their actual age at menarche, sampling error is 5%, confidence interval of 95%, and the significant level t-value at alpha level of 0.05 is 1.96, the calculated return sample size without estimated response factor was 384. With the study population of 990, and an estimated response rate of 70%, the drawn sample size of 389 was obtained for this study. The number of respondents from each class in a study programme was obtained using a proportional approach based on the number of female students in the class. In each class, respondents were randomly chosen by picking from an envelope containing pieces of paper with names and identity number of each female member of the class printed on it. The respondents were drawn using the sampling with replacement method.

### Menarcheal age measurement

The menarcheal age of respondents was determined using the recall method. Respondents were requested to state to the nearest whole year, how old they were when they first experienced menstrual flow.

### Statistical analysis

Data were entered into Microsoft Excel, and analyzed using Graph Pad Prism, Version 5.01 (GraphPad Software Inc., San Diego CA). Associations between respondents’ socio-demographic characteristics and age at menarche were assessed using the independent t-test. Statistical significance was assumed at p < 0.05 and a confidence interval of 95%.

## Results

### Socio-demographic profile

The socio-demographic profile of the respondents is as shown in Table 1. In this study, the majority, 221 (75.4%) were between ages 20 and 25 years (Mean age ± standard deviation = 23±5.07; Median = 22 years), Christians, 208 (71.0%), and spent their vacations in urban areas of Ghana, 181 (61.8%). At menarche, most respondents, 126 (43.0%) stayed in a self-contained accommodation indicative of their parents and guardians belonging to the middle social class. Most respondents, 99 (33.8%) were studying nursing.

**Table 1:** Socio-demographic profile of the respondents.

Variable	Subgroups	Number of respondents	Percentages
Age (years)	< 20	33	11.3
	20–25	221	75.4
	> 25	39	13.3
Religious affiliation*	Christianity	208	71.0
	Islam	79	27.0
Course of study	Community Nutrition	52	17.7
	Health Science Education	29	9.9
	Medicine	54	18.4
	Midwifery	59	20.1
	Nursing	99	33.8
Type of accommodation at menarche occupied by her family*	Single room	46	15.7
	Chamber and hall	55	18.8
	Several rooms in a compound house	52	17.7
	Self-contained apartment	126	43.0
	Mansion	10	3.4
Place of residence during vacation*	Urban area	181	61.8
	Sub-urban	88	30.0
	Rural	21	7.2

*There were missing values so percentage did not add up to 100.

### Menstruation history and characteristics of respondents

Majority of respondents, 161 (54.0%) had their age at menarche between 13 and 15 years (Mean ± standard deviation = 13.66±1.87; Median = 14 years); 195 (66.6%) began to menstruate when they were in junior high school, 212 (72.4%) had regular menstrual pattern, and 245 (83.6%) experienced moderate menstrual flow. For most, 102 (34.8%) respondents, it took 4 days, for the menstrual flow to stop (Mean± standard deviation = 4.484±1.244; Median = 4 days). The overall prevalence of dysmenorrhea in this study was 83.6% (n = 245). The menstrual pain began before the menstrual flow for, 143 (58.4%) of respondents with, 184 (62.8%) experiencing menstruation associated symptoms. The gynaecological age of the respondents was 9.375±4.7 (Range 2 to 33 years). Table 2 shows the menstrual history and characteristics of respondents.

**Table 2:** Menstruation history and characteristics of the respondents.

Variable	Subgroup	Number of students	Percentage
Age at menarche	<13	83	28.3
	13–15	161	54.0
	>15	49	16.7
Menstrual pattern	Regular	212	72.4
	Irregular	70	23.9
	Don’t know	11	3.8
Nature of menstrual flow	Light	9	3.1
	Moderate	245	83.6
	Heavy	38	13.0
Number of days of menstrual flow	2	9	3.1
	3	47	16.0
	4	102	34.8
	5	77	26.3
	>6	50	17.1
Class you started menses	Primary	68	23.2
	Junior high school	195	66.6
	Senior high school	25	8.5
Presence of dysmenorrhea	Yes	245	83.6
	No	48	16.4
	Before blood begins to flow	143	58.4
	During the menstrual flow	98	40.0
	After blood had stopped	0	0.0
Presence of other associated symptoms?	Yes	184	62.8
	No	85	29.0

### Distribution of the menarcheal ages among the respondents

**Fig 1:**
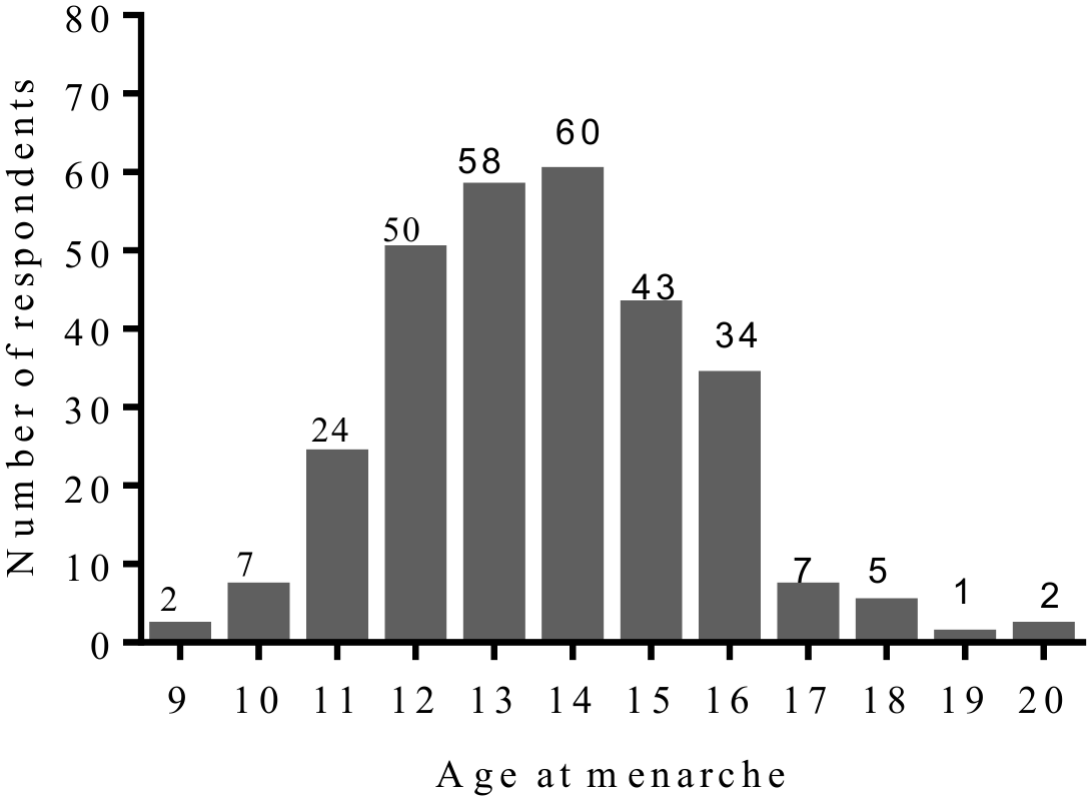
Distribution of the recalled menarcheal ages of respondents. Number above each bar represents the actual number of respondents for each stated recalled age at menarche.

Fig 1 shows the lowest age at menarche was 9 years with the highest being 20 years. The modal menarcheal age was 14 years, 25^th^ percentile was 12 years and 75^th^ percentile, 15 years (95% CI = 13.45–13.88).

### Effects of socio-economic differences and course of study on the age of menarche of respondents

Table 3 shows the effect of social status of parents on age at menarche of respondents as measured by the type of accommodation occupied at menarche and living areas of the respondents. There were statistically significant differences in menarcheal ages based on the type of accommodation occupied at menarche (χ^2^ = 23.19, df = 8, p = 0.003). None of the respondents who lived in a mansion (mean menarcheal age = 13.11±1.054 years) had menarche after 15 years compared to between 10.3% and 30.8% for other categories. Students who resided in urban areas of Ghana recorded the lowest menarcheal age (13.41±1.709 years). Proportion of students based on living area whose age at menarche was greater than 15 years were; urban (13.8%), sub-urban (17.0%) and rural (47.4%). The differences of menarcheal age based on living area was statistically significant (χ^2^ = 14.51, df = 4, p = 0.006). Differences in age at menarche between students based on the course of study was significant (χ^2^ = 31.55, df = 8, p = 0.0001) with medical students experiencing the lowest age at menarche (13.15±1.295 years) and the midwifery class, the highest of 14.47±2.269 years.

**Table 3:** Distribution of ages at menarche based on type of accommodation and living area at menarche as well as course of study of respondents.

Variable	Subgroup	Ages of menarche (years)			Mean ages (years)	χ^2^, (df)	p-value
		<13	13–15	>15			
Type of accommodation at menarche	Single room	11 (23.9)	27 (58.7)	8 (17.4)	13.74±1.769		
	Chamber and a hall	10 (18.2)	34 (61.8)	11 (20.0)	14.11±1.808	23.19 (8)	0.003*
	Several room in a compound house	8 (15.4)	28 (53.8)	16 (30.8)	14.52±1.925		
	Self-contained apartment	49 (38.9)	64 (50.8)	13 (10.3)	13.20±1.762		
	Mansion	3 (30.0)	7 (70.0)	0 (0.0)	13.11±1.054		
Home living area	Rural	3 (15.8}	7 (36.8)	9 (47.4)	14.76±2.364		
	Sub-urban	22 (25.0)	51 (58.0)	15 (17.0)	13.88±1.976	14.51 (4)	0.006*
	Urban	56 (30.9)	100 (55.2)	25 (13.8)	13.41±1.709		
Course of study	Health Science Education	6 (20.7)	17 (58.6)	6 (20.7)	14.00±1.512		
	Community Nutrition	15 (28.8)	25 (48.1)	12 (23.1)	13.88±2.016		
	Midwifery	12 (20.3)	26 (44.1)	21 (35.6)	14.47±2.269	31.55 (8)	0.0001*
	Nursing	34 (34.3)	56 (56.6)	9 (9.1)	13.24±1.709		
	Medicine	16 (29.6)	37 (68.5)	1 (1.9)	13.15±1.295		

* Statistically significant.

### Relationship between menarcheal age and menstrual characteristics of respondents

Table 4 shows the relationship between menarcheal age and the menstrual characteristics of respondents. The age at menarche of respondents does not seem to define the menstrual characteristics such as type of menstrual cycle, presence or absence of dysmenorrhea, severity of menstrual pain and presence or absence of menstruation associated symptoms exhibited by females. This study however showed that age at menarche has a significant effect on when menstrual pain begins in a cycle for females who suffer dysmenorrhea (χ^2^ = 7.181, df = 2, p = 0.028). Females who experience menstrual pain before menstrual flow had earlier age at menarche than those who experience the pain during blood flow (13.52±2.052 vrs 13.63±1.582, p = 0.028).

**Table 4:** Relationship between menarcheal age and menstrual characteristics of respondents.

Variable	Subgroup	Mean age	Age at menarche (years)			χ^2^, (df)	p-value
			<13	13–15	>15		
Type of menstrual cycle	Regular	13.74±1.919	59 (74.7)	115 (74.2)	38 (79.2)	0.500 (2)	0.779
	Irregular	13.49±1.717	20 (25.3)	40 (25.8)	10 (20.8)		
Presence of dysmenorrhea	Yes	13.58±1.862	74 (89.2)	129 (80.1)	42 (85.7)	3.451 (2)	0.178
	No	14.08±1.877	9 (10.8)	32 (19.9)	7 (14.3)		
Severity of pain	Mild	14.08±1.770	13 (17.6)	16 (13.4)	13 (31.0)	6.689 (4)	0.153
	Moderate	13.51±1.888	43 (58.1)	75 (63.0)	20 (47.6)		
	Severe	13.29±1.822	18 (24.3)	28 (23.5)	9 (21.4)		
When pain begins	Before blood flow	13.52±2.052	51 (68.9)	64 (51.2)	28 (66.7)	7.181 (2)	0.028*
	During blood flow	13.63±1.582	23 (31.1)	61 (48.8)	14 (33.3)		
Type of menstrual flow	Light	14.11±1.364	2 (2.4)	6 (3.7)	1 (2.0)	2.784 (4)	0.595
	Moderate	13.76±1.890	66 (80.5)	135 (83.9)	44 (89.8)		
	Heavy	13.03±1.716	14 (17.1)	20 (12.4)	4 (8.2)		
Presence of associated symptoms	Yes	13.53±1.727	54 (68.4)	103 (71.5)	27 (58.7)	2.656 (2)	0.265
	No	13.81±2.168	25 (31.6)	41 (28.5)	19 (41.3)		

*Statistically significant.

## Discussion

This study found that the majority of respondents experienced menarche between ages 13 and 15, with a mean recall age at menarche of 13.66±1.87 years. Females, who came from more affluent homes, lived in urban areas and were studying medicine experienced menarche earlier than those from poorer homes, dwelt in rural areas and were pursuing non-medical programmes. Females who experienced menstrual pain before menstrual flow had earlier age at menarche than those who experienced the pain during blood flow.

With the mean biological age of respondents who had all experienced menarche being 23±5.07, the recall method of determining the age at menarche is the most appropriate and reliable. The accuracy of the recall method was shown in a study by Must, Phillips, Naumova, Blum, Harris et al. (2002) which reported that even 29 years after menarche, there was a high correlation between the actual menarcheal age and the recalled menarcheal age among women [21]. With a mean recall gynecological age (current age less age at menarche) in this study being less than 10 years (9.375±4.728), the use of the retrospective method in this study should provide an accurate age at menarche for females in Ghana.

The mean menarcheal age of 13.66±1.87 years found in this study was a little above the overall mean age of menarche of 13.53±0.98 years reported in a survey of 67 countries in 2001 [21] but lower than 13.98±1.42 years Adadevoh et al. reported in 1986 which was the first major survey to find the age at menarche of Ghanaian girls. The age at menarche in this study is however lower when compared to reports from some other African countries such as Mozambique, Ethiopia and Nigeria [22], [23], [24], [25]. Countries from which age at menarche was reported lower than that found in this study include US, Canada, Italy, Portugal, and Turkey [1], [3], [8], [13], [26], [27], [28]. Other studies in Ghana that also used the recall method; Aryeetey, Ashinyo and Adjuik (12.74±1.15) and Gumanga and Kwame-Aryee (12.5±1.28) all reported lower age at menarche in Ghana [20], [29]. These two studies in Ghana however used subjects who were in junior or senior high schools whose ages would normally not be above 18 years. With 2.7% of respondents in this study recording age at menarche of 18 years and above, this study captured females who experience late menarche which is possibly the reason for the difference in the mean menarcheal age obtained in this study and those reported by Aryeetey et al. and Gumanga & Kwame-Aryee [20], [29]. Using the mean menarcheal age reported by Adadevoh et al., (1989) as baseline, the decline of mean menarcheal age per decade for this study is 0.11 year per decade. Similar decline rate of 0.1 year per decade was observed in Italy [12]. This declining age at menarche in Ghana is of public health concern as there is a potential for increased incidence of non-communicable diseases such as hypertension, diabetes mellitus, coronary heart disease and breast cancer among Ghanaian women because various studies have shown associations between these diseases and early menarche [14], [15], [16], [17], [18]

The effect of socio-economic circumstances on the age of menarche has been shown in several studies with girl children in more deprived situations experiencing later menarche as they are unable to obtain the appropriate nutrition for proper growth and development [5], [9], [10], [12], [30]. This study also found that females who reside in rural areas and whose parents belong to lower socio-economic levels experience significantly delayed menarche when compared with urban dwellers and those of middle or high socio-economics status. This study further found significant differences in age at menarche among students based on the courses they pursue at this university; with the medical students experiencing the earliest menarche. This may be attributed to the situation in Ghana where only students who score the best grades enter medical schools to pursue medicine and these grades are best made by students who live in the cities and whose parents are above the lower social class. More than three fourth of the medical students in this study live in urban areas of Ghana and hence the possible reason for them recording the lowest age at menarche would be the higher socio-economic class of their parents. This study showed that females who experienced early menarche were more likely to begin experiencing menstrual pain before menstrual flow begins. Harlow and Park, (1996) reported that earlier age at menarche increases the occurrence, duration and severity of menstrual pain [31]. This means, with declining age at menarche, there is increased morbidity due to dysmenorrhea, with more disruptions in the daily activities of post-pubescent females. With possible increase in incidence of dysmenorrhea in the future, it would become a condition of greater public health concern.

Despite these interesting findings, this study has several limitations that may affect its broad applicability. It was undertaken among female students of the Tamale campus of the University for Development Studies due to limited resources. The results of this study can therefore not be generalized for all females in Ghana. Measuring ages at menarche using retrospective or recall method is said to be influenced by error of poor memory unlike the status quo and prospective methods [9], [12]. However, since all respondents in this study had already experienced menarche, the recall technique was the only feasible method. Despite the limitations of the retrospective method such as memory bias, several studies have shown that measuring ages at menarche by recall especially when the study is done close to the menarcheal episode, has a high level of accuracy [12], [32], [33]. The use of simple random sampling minimized the biases in relation to the selection of respondents which is one of the strengths in this study. Furthermore, the level of education of the respondents and the fact that they were in school when menarche occurred makes their recall age at menarche more reliable.

## Conclusion

The mean age of menarche of the respondents in this study was 13.66±1.87 years when most of them were in the junior high schools. Age at menarche was influenced by socio-economic status of the girl child and their families as females who lived in urban areas and from higher socio-economic environment, experienced early menarche than those from rural and lower income situations. Lower menarcheal age was associated with an earlier onset of menstrual pains in dysmenorrhic females. Compared to the first Ghanaian study in 1986, the decline per decade of menarcheal age of 0.11year recorded in this study may explain Ghana’s increasing burden of non-communicable diseases which are associated with early menarche. The declining age at menarche in Ghana may also reflect an upward trend in better living standards in Ghana as declining mean menarcheal age is strongly correlated with improved socio-economic development.
